# Supplementary material for: New Insights of Potassium Sources Impacts as Foliar Application on ‘Canino’ Apricot Fruit Yield, Fruit Anatomy, Quality and Storability
Source: Plants (Basel). 2021 Jun 8;10(6):1163. doi: 10.3390/plants10061163 (PMC8229475; doi:10.3390/plants10061163)
Supplement: Supplementary file 1 [file plants-10-01163-s001.zip › plants-1199498-supplementary.pdf]

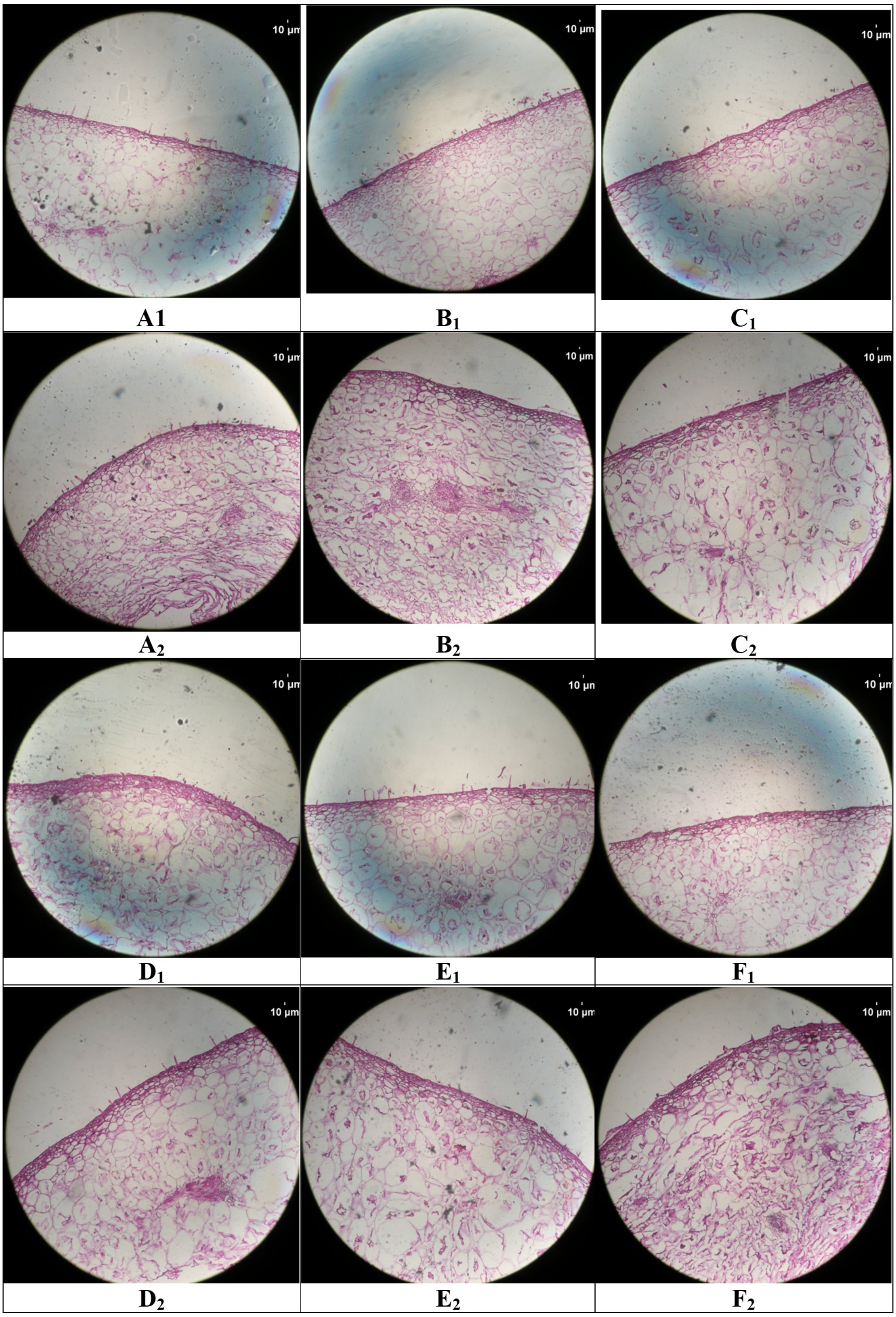

Figure S1: The effect of potassium sources on the cuticle, parenchyma cell and cell wall thickness of Canino apricot fruit tissue at harvest (A_1,B_1,C_1,D_1,E_1,F_1) and at the end of the storage period (A_2,B_2,C_2,D_2,E_2,F_2) and bar = 10 µm.. The letters given next to each photo belongs to the treatments in the order of control (A_1 and A_2), k-Sulphate (B_1 and B_2), k-nitrate ($C_1$and C_2), k-citrate (D_1 and D_2), k-silicate (E_1 and E_2) and k-humate (F_1 and F_2) treatments, at harvest and at the end of the storage, respectively.(A_2,B_2,C_2,D_2,E_2,F_2) and bar = 10 µm.. (A_2 and F_2 show that the damage at end of cold storage was more evident in the mesocarp sub- epidermal tissue of the control and k-humate fruits, respectively).
